# Can “Free-From” Food Consumption Be a Signal of Psychological Distress during COVID-19?

**DOI:** 10.3390/foods11040513

**Published:** 2022-02-11

**Authors:** Mariarosaria Savarese, Greta Castellini, Lorenzo Morelli, Guendalina Graffigna

**Affiliations:** 1 EngageMinds HUB—Consumer, Food & Health Engagement Research Center, Università Cattolica del Sacro Cuore, 20123 Milan, Italy; mariarosaria.savarese@unicatt.it (M.S.); guendalina.graffigna@unicatt.it (G.G.); 2 Faculty of Agricultural, Food and Environmental Sciences, Università Cattolica del Sacro Cuore, 26100 Cremona, Italy; Lorenzo.morelli@unicatt.it; 3 Institute of Microbiology, Università Cattolica del Sacro Cuore, 26100 Cremona, Italy

**Keywords:** clean eating, consumer psychology COVID-19, free-from food consumption

## Abstract

During the last year, feelings of anxiety and depression were registered among the Italian population and affected food consumption. Among the research that explored people’s dietary inclinations during the current pandemic, no previous studies have explored psychological factors associated with the “free-from” dietary pattern. Our study is aimed at understanding if free-from food consumption orientations can be associated with negative psychological distress. We conducted a web-based survey between 27/10/2020–03/12/2020 on a representative sample of 963 Italians. Psychometric scales and ad hoc items were used to measure people’s levels of anxiety, depression, fear for contagion and consumption orientation towards “free-from” foods. Of the sample, 18.2% frequently purchased gluten-free food products and 22.5% purchased lactose-free foods. Most of the population (44.1%) feels very at risk of contagion from COVID-19 and suffers from anxiety (52.8%) and depression (55.0%). Free-from consumers are more anxious, depressed, have higher risk of contagion, and are younger than the non-consumers. During the COVID-19 pandemic, free-from foods can represent for the people a way to restore control over their lifestyle, which was denied during the emergency. However, we highlight possible negative long-term effects of this dietary choice.

## 1. Introduction

During the last year, the COVID-19 pandemic led to deep lifestyle changes all over the world, especially in the most hit countries, such as Italy. The alternation of lockdowns and the slackening of the restrictions—based on the epidemic situation—brought the population continuous psychological ups and downs [1,2,3]. People experienced uncertainty about the duration of the quarantine, fear of getting infected or dying, for themselves or their loved ones, frustration, boredom, infodemic, overall uncertainty of the future, fear of significant financial losses, or long-term repercussions on the country [4,5]. These events put a strain on the emotional sphere of every individual, causing anxiety and depression. Research carried out by Ammar and colleagues during the COVID-19 outbreak on over a thousand people in home confinement reported a reduction in mental well-being and satisfaction and an increase in depression and need for psychological support, compared to the pre-epidemic period [6]. Results from an Italian study showed that 38% of the population was affected by significant psychological distress [7]. A study conducted in China monitored depression and anxiety levels of the population in the first period of the COVID-19 emergency, highlighting an increase in these psychological outcomes among the population [8]. Similar results were also supported by a systematic review and meta-analysis published by Salari et al. [9]. The perception of psychological distress, moreover, becomes particularly relevant in emotionally sensitive periods such as Christmas, when people in Italy—and all over the world—are used to spending more time with their families and friends, especially around the table [10]. Between November and December, the contagion rate In Italy was still around 20,000 cases per day [11]. The Country was divided into three zones (yellow, orange, and red) referring to a complex contagion index, with most of the regions in the middle level of risk. The attention of the Italian Government was focused on planning the preventive measures to avoid the increase of contagions during Christmas Holidays, as the disease burden on the Health Care System was still high and uncontrolled. As a consequence, people were experiencing very controversial feelings: on the one hand, they were conscious of the very precarious epidemic situation; on the other they demonstrated frustrations and fatigue in expecting to spend this particular moment of the year in solitude. These controversial circumstances generated psychological repercussions, increasing levels of anxiety and frustration which, in turn, may have had an impact on their orientation towards food. The mutual association between diet and psychological state is indeed well known [12,13], in particular in the way low mood and poor nutrition are connected [14]. Several studies highlighted that a significant percentage of individuals changed dietary habits during the COVID-19 pandemic, due to its physical and psychological impact. According to recent studies, staying at home, smart working, and limited physical activity, together with consequent boredom and stress, have led to emotional eating [15,16], expressed especially through the consumption of ‘comfort foods’, higher in energy and richer in sugar or fat [17,18]. Another study on a sample of 602 Italians reported that almost half of the respondents felt anxious due to the forced change in their eating habits, consumed comfort food and were inclined to increase food intake to feel better [19]. In addition, limited access to daily grocery shopping may have led the population to reduce the consumption of fresh foods, especially fruit, vegetables and fish, in favor of highly processed and long shelf life products [20]. In order to cope with the negative experience of self-isolation, people have been more prone to look for reward and gratification physiologically associated with those food consumptions [21]. Those foods, indeed, can reduce negative emotional states as they encourage serotonin production with a positive effect on mood, but they are also associated with the increased risk of developing obesity and cardiovascular diseases [22].

On the other hand, negative psychological experiences may also have led to voluntarily eating restrictions, due to the psychological distress, to the fear of embracing unhealthy conduct or to compensate the forced lack of physical activity. Among Western consumers, indeed, there is increasing adoption of special diets that fall under the umbrella of “clean eating”. At different levels, these diets represent people’s extreme orientation toward healthy food, and obsessively control one’s own weight, also in response to psychological distress, anxiety or routine restraints [23,24,25]. These diets typically include elements such as eating local, “real” (non-processed), organic, plant-based, home-cooked foods [26], but often also tout more extreme strategies such as eliminating gluten or lactose [27]. Gluten- and lactose-free diets are nowadays food selection strategies with rising popularity also in Italy. Originally supposed to simplify intolerant people’s daily food choices, they are now spread among the entire population as one of the most increasing food trends of the latest years. Despite their popularity, this trend hides potential risks when taken to the extreme, both for public health and consumers’ attitudes towards their diets, especially in a country like Italy where it risks turning people away from a more balanced diet such as the Mediterranean one. In addition, COVID-19 fallouts may have enhanced people’s tendency to compensate the lifestyle restrictions and emotional distress with inadequate food choices.

In this framework, it is clear that the food selection is based on psychological and emotional aspects, and that the COVID-19 pandemic could have been exacerbated some unbalanced food trends. Although it is easy to find an increasing body of literature that focuses on the dietary inclinations of the current pandemic towards junk food or comfort food [28], to the best of our knowledge, no previous studies have explored psychological factors associated with the restrictive or clean dietary patterns in Italy during the COVID-19 emergency, where people have particularly suffered from psychological burden. For these reasons, our study is aimed at understanding if free-from food consumption orientations can be associated with negative psychological distress.

## 2. Materials and Methods

### 2.1. Sampling and Materials

Research data were collected via a questionnaire that was filled out by a representative sample of the Italian population with sex, age, profession, size of the centre, and geographical area extracted by stratified sampling. The percentages relating to the Italian population were taken from the website of ISTAT [29] and reported in Table 1. The survey was conducted using a CAWI (Computer Assisted Web Interviewing) methodology between 27 October–3 December 2020 to catch consumers’ orientation in anticipation of Christmas, as a particularly sensitive time of the year, and in prevision of the more restrictive measures in planning for the holidays. The sample consists of 1005 subjects randomly selected from the consumers’ panel managed by Norstat srl [30] from which 42 people were removed as intolerant to lactose and/or gluten, generating a final sample of 963 subjects. This study is part of wider research that investigates how COVID-19 disease influences the lifestyles, concerns and food consumption of the Italian population. This study has been performed following the Declaration of Helsinki and has been approved by an independent ethics committee of Università Cattolica del Sacro Cuore in Milan (CERPS).

The survey included:

- A series of questions regarding participants’ socio-demographic profiles. In particular, the variables included in the present study were:
  ◦ Gender;
  ◦ Age: participants were clustered into three groups, namely young adults (18–38), middle-age (39–53), and over 54;
  ◦ Monthly net family wage: participants were clustered in two groups, namely above and below the median wage in the sample of 1800 Euros;
  ◦ Education, with participants grouped as having a university degree or not.
- Psychological variables:
  ◦ Anxiety symptoms were assessed with the Self-rating Anxiety Scale (SAS) [31] and the depression symptoms with the Self-rating Depression Scale (SDS) [32]. Each scale contains 20 questions, and the respondents were required to indicate the frequency they felt the emotions during the last week on a 4-point scale (1 = very little time or not at all; 4 = all the time). For the anxiety scores ≤ 35 were considered normal, 35 < scores ≤ 47 were considered to indicate mild anxiety, 47 < scores ≤ 59 were considered to indicate moderate anxiety, and scores > 59 were considered to indicate severe anxiety [31]. For the depression scores ≤ 39 were considered normal, 39 < scores ≤ 47 were considered to indicate mild depression, 47 < scores ≤ 55 were considered to indicate moderate depression, and scores >55 were considered to indicate severe depression [32].
  ◦ In order to measure the risk susceptibility of contagion from COVID-19, the participants were asked their perceived risk of being infected by the new COVID-19 virus using a 5-point Likert scale from 1 (very little) to 5 (a lot).
- Consumers’ orientation towards lactose-free and gluten-free food products: was assessed by two separate questions that evaluated the self-reported consumption frequency of these products in the last month, in line with other research [33]. The responses were assessed on a 5-point Likert scale from 1 “never purchased” to 5 “always purchased”. The two items used were: “In the last month, how many times have you purchased lactose-free food products?”; “In the last month, how many times have you purchased gluten-free food products?”. These questions about food consumption had “I do not know” as an option of answer. The subjects who chose this answer were considered as “missing data” and, therefore, not reported within the analysis in order to obtain more readable results.

### 2.2. Data Analysis

The data obtained were analyzed using descriptive statistics and calculating frequencies, percentages, averages, and standard deviations. To analyze the associations between the frequency of purchase of lactose-free and gluten-free food products, sociodemographic characteristics (sex, age, level of education, and income level) and the psychological features (level of anxiety, depression, and perception of risk from COVID-19 infection), a series of Pearson’s and chi-square correlations were carried out. Moreover, contingency tables and One-way ANOVAs, followed by Bonferroni’s post-hoc comparisons, were applied (after verifying that the distribution of the dependent variables and their residuals were normal) in order to understand the psychological and sociodemographic differences between four groups: those who purchased in the last month both lactose-free and gluten-free food products, those who purchased gluten-free foods more frequently, those who purchased lactose-free foods more frequently and those who did not purchase the two products in the last month. To create these groups, we used the two questions regarding the consumption of gluten-free and lactose-free products and through the filter functions of SPSS, we created these four groups. In particular, when the dependent variables, used in the ANOVAs, violated the assumption of homoscedasticity, Welch’s ANOVA was preferred over a classic ANOVA approach to provide a more robust method for data analysis [34]. All analyses have been carried out with IBM SPSS 20 (release 20.0.0.0).

## 3. Results

The sample is composed of 963 people of which 478 are male and 485 are female, aged between 18 and 70 years (M = 45.44, SD = 14.1). The demographic profile is presented in detail in Table 1.

Concerning the consumption of gluten-free and lactose-free food products, the results show that 18.2% of the sample purchased frequently, in the last month, gluten-free food products (4 and 5 points on likert scale) while the majority of the population (43.5%) have never bought them (Table 2). As for lactose-free products, 22.5% of the sample frequently consumed these food products (4 and 5 points on likert scale), a percentage significantly higher than the consumption of gluten-free products (*p* < 0.01), while 39.5% declared to have never consumed lactose-free food products in the last month (Table 2). Comparing the average consumption of lactose-free and gluten-free food products, the results show that the Italian population purchased lactose-free products more frequently than gluten-free ones (2.18 vs. 2.29, *p* < 0.01).

Regarding the psychological variables, most of the population (44.1%) feels very at risk of contagion from COVID-19 while only 16.2% declare a low risk of contagion. Considering the levels of anxiety and depression, results underline that more than half of the Italian population suffers from moderate, mild, or severe anxiety (52.8%) and depression (55.0%) (Table 3).

As for the associations between the consumption of gluten-free and psychological variables, the results show that higher levels of anxiety, depression, and risk susceptibility are associated with a more frequent gluten-free food consumption (respectively, r = 0.389, *p* ≤ 0.001; r = 0.286, *p* ≤ 0.001; r = 0.135, *p* ≤ 0.001). Moreover, men (Chi-square = 5.240 (df = 1), *p* < 0.05) with a high income (Chi-square = 6.571 (df = 1), *p* < 0.05) and high level of education (Chi-square = 4.556 (df = 1), *p* < 0.05) consume gluten-free food products more frequently. Furthermore, age is negatively correlated with this consumption (r = −0.183, *p* ≤ 0.001), highlighting how the youngest are those who most frequently consume gluten-free products.

Regarding the lactose-free food consumption, the results show that, in this case, higher levels of anxiety, depression, and risk susceptibility are associated with a more frequent lactose-free food consumption (respectively, r = 0.340, *p* ≤ 0.001; r = 0.249, *p* ≤ 0.001; r = 0.141, *p* ≤ 0.001). Moreover, those with a high income (Chi-square = 7.267 (df = 1), *p* ≤ 0.01) consume lactose-free food products more frequently. Furthermore, age is negatively correlated with this consumption (r = −0.163, *p* ≤ 0.001), highlighting how the youngest are those who most frequently consume lactose-free products. On the contrary, there are no differences related to gender and level of education.

After verifying the normality of the dependent variables introduced in the analyses (see Table 3) and the normality of their residues (Kurtosis ranges from −0.93 to 0.91 and Asymmetry from −0.35 to 0.54), the study deepened the differences between the four groups of gluten-free and lactose-free consumers: those who consume both gluten-free and lactose-free food products, those who purchase lactose-free products more frequently, those who buy gluten-free products more frequently, and those who do not consume these products (Table 4). Regarding the socio-demographic differences, the results show that the groups differ in age. In particular, those who buy gluten-free more frequently (group 3) are younger than other groups, while those who do not purchase “free-from” foods (group 4) are older. There are no differences between groups in level of education, gender, and level of income. Regarding psychological differences, results show that the levels of anxiety (F[3, 414.68] = 34.552; *p* ≤ 0.001, η^2^ = 0.10), depression (F[3, 917] = 23.827; *p* ≤ 0.001, η^2^ = 0.07), and risk susceptibility (F[3, 917] = 5.898; *p* ≤ 0.01, η^2^ = 0.02) are associated with different types of consumption. In particular, those who do not consume free from food products (group 4) have significantly lower levels of anxiety than the other groups, and those who buy both products (group 1) or prefer the purchase of gluten-free foods (group 3) have higher levels of anxiety. Regarding the levels of depression, the results show that those who buy both lactose-free and gluten-free foods (group 1), or at least one of them (2 and 3), have a very similar level of depression, but significantly higher than those who do not consume them (group 4). Regarding the levels of risk perception from COVID-19, the results underline that there is a significant difference between those who purchase both “free-from” (group 1) products and those who do not buy them (group 4) and in particular the latter feels much less at risk of contagion than the former. Briefly, it is possible to affirm that there are no significant differences between those who buy lactose-free, gluten-free products, or both foods (groups 2, 3, and 1), but substantial differences are observed between those who purchase “free-from” food products and those who do not consume them. In particular, the latter (group 4) are less anxious, depressed, with a lower risk of contagion, and older than the other groups.

## 4. Discussion

Among the aftermaths that the COVID-19 emergency is leaving behind, particular attention has been given to consumers’ psychological reactions towards the food. The restrictive measures, necessary for the containment of the virus, had a profound impact on people’s lifestyle, and, consequently, on their way of approaching food consumption [17,22,35]. In Italy, in particular, the citizens spent most of the past year, and the beginning of 2021, in home confinement, and they experienced feelings of anxiety, depression, and social isolation, which led them to pour out their negative emotions on the food choices [20,36]. Moreover, at the beginning of December 2020, people in Italy lived a particularly controversial period swinging between the hope of spending the Christmas holidays with their loved ones and the awareness that the epidemic situation was still too risky to allow it. The mitigation strategies called by the Government—necessary to limit the contagions and the burden on the Health Care System—had a profound psychological impact on the individuals and their orientation towards food consumption. Scholars registered higher consumption of junk and comfort food, traditionally defined as food to cope with negative emotions, boredom, or feelings of uncertainty [37]. In our study, we proposed a new view on this phenomenon, as we tried to understand whether a “clean food choice” could also be used as a sort of compensation for psychological distress. Indeed, we investigated the intention to consume gluten and lactose-free foods in Italy, and we explored the relationship between these consumptions and feelings of anxiety, depression, and risk perception among the population.

Results showed that at the end of 2020, in Italy, around 20% of the population declared to have purchased gluten- or lactose-free foods, while the majority of the population did not purchase them. These results have been cleansed from all the subjects who reported diagnosed intolerance, to highlight the deviation between the number of people who really needed these foods for medical reasons and the ones who approached this consumption as a voluntary choice. In this framework, this percentage appears of note, and in line with recent trends [38]. The “clean eating” movement, especially the consumption of gluten- and lactose-free foods was investigated during the last 10 years, and the results showed a surprising increase in the consumption of these products [39].

Our results also allowed us to draw a psychological portrait of the Italian population coping with COVID-19 in terms of feelings of anxiety, depression, and uncertainty for risk of contagion. Similarly to other data collection conducted on the same territory [40,41], the percentage of people experiencing feelings of anxiety and depression are nearly half of the population, with 44.1% feeling very at risk of contagion from COVID-19. Not surprisingly, people in Italy are experiencing a poorer quality of life since the COVID-19 emergency hit the country in January 2020. Similar results underline the need to consider COVID-19 more as a syndemic [42], which means that the consequences of the health emergency cannot be separated from the contextual, such as economic and social elements that characterize a country [43]. In Italy, in particular, the already fragile and precarious social and political conditions enhanced the impact of the health emergency and led the population to a state of psychological and mental frustration and distress. Even if anxiety can be considered a natural emotional state in response to what people think to be dangerous [44], in the time of the COVID-19 is important not to neglect its potential long-term impact. The common belief that the negative collective emotional states are a natural heritage of this pandemic should not distract professionals and governmental bodies from providing adequate support for the people who are and will be in need.

Peculiarly, our study put in relation people’s emotional state with the consumption of gluten- and lactose-free foods, in order to explore possible connections. Our results show that higher levels of anxiety, depression, and risk susceptibility are associated with more frequent gluten- and lactose-free food consumption. In addition, those results are strengthened by the fact that—in our sample—significant differences between “free-from” consumers and the non-consumers were found, with the first ones having been found more psychological fatigued by the COVID-19 pandemic. These results have some points of discussion compared to what has been published until now. It is known, indeed, that carbohydrate-rich foods are used as a way of self-medicating depression, as they tend to raise serotonin levels thus lowering depression symptoms [45]. Many studies, indeed, claimed that during COVID-19 lockdowns people had more episodes of “food craving”, especially for richer and tastier food [46]. Moreover, in Italy, during the past year, scholars highlighted the risk for the population to undertake inadequate diets, thus having negative long-term consequences on their health status [47]. Our results suggested, instead, that people who consumed gluten- and lactose-free foods had a higher level of psychological distress, thus underling their fragile emotional state. It seems that—as happened with comfort foods— “free-from” foods were also used in some way as a strategy to cope with the negative fallouts of the COVID-19 emergency. What differs, however, is the idea that free-from foods are not typically described as comfort or rich foods, but more as healthy, pure, and simple [48]. This trend seems to be in countertrend to what has been studied until now where emotional eating was mainly expressed through flavorful and pleasant foods that the consumers used to compensate their unbalanced emotional state. In this case, it seems that the “free-from”, in other terms, the idea to proactively decide to eliminate an ingredient from the diet, gave the consumer a sense of control and personal power that was lacking in the uncertainty of the pandemic, especially for those with poorer mental wellbeing. People with higher levels of anxiety and depression were also demonstrated to be more suspicious towards some nutrients, just for the fact that they were unnecessary and could be eliminated from the specific food. As happens in the “clean eating” movement, where people voluntarily decide to limit their diets in order to control their intake, in our case they express the need to take the control of the diet as something that is controllable in an uncontrollable context. These results resound some studies on the concepts of restrained eating and orthorexia nervosa, which were considered in similar cases such as veganism and vegetarianism [49,50]. These diets are all characterized by the reduction of food intake according to specific criteria with the intention to control one’s weight [50]. While it is a reasonable strategy for people who are overweight, it is risky for individuals who are normal or underweight and could be, therefore, a risk factor for eating disorders [49]. Even if preliminary, our results can suggest that the state of “emotional pandemic” related to the COVID-19 health pandemic could rise in the population a sense of disorientation and lack of control, which can be poured on the food consumption. This is—on the contrary—a personal sphere where people can establish their control, as food is a deliberate and personal choice [51].

Finally, our study also defined a demographic profile of people who were more oriented to “free-from” consumption, who—in general—are younger, in line with what the past literature also reported [52]. Gluten-free consumers had a higher level of education, while lactose-free consumers had a higher income. Furthermore, these results confirm past research [53]. In our sample, a higher percentage of males consumed gluten-free products, which is different to what other studies report [52].

Despite these novel results, our research has some limitations. We investigated “free-from” consumption with self-reported measures, which can be susceptible to cognitive and memory bias. However, having the aim to study consumers’ personal representation of this phenomenon, we preferred to adopt this strategy. We also analyzed the consumption of general gluten- and lactose-free foods, with no precise reference to a specific product. This could be a limitation, but by making this choice we wanted to give relevance more to the idea of the “free-from” trend that anchor to a specific product, which could have limited consumers’ answers. In addition, no explicit measures around the topic of restrained eating or orthorexia nervosa were used, which could have added strength to our results. We preferred to discuss this topic from a consumer psychology perspective, which means not falling into clinical recommendations. Our intent is to give a deep explanation of a market trend that—until now—still shows some controversial aspects in terms of consumer attitudes and perceptions. We suggest considering these aspects in future research in order to address our research gaps.

## 5. Conclusions

The COVID-19 emergency gave us the grounding text to confirm the role of the psychological dimensions in orienting consumers’ orientation towards food consumption. In the Italian context, free-from food consumers reported poorer mental wellbeing (in terms of higher levels of anxiety, depression, and fear of the contagion) compared to non-consumers, highlighting that these foods can represent, for them, a way to restore control over their lifestyle, which was denied during the emergency. In this scenario, however, we highlight possible negative long-term effects of this dietary choice. Not understanding the deep motivation behind extreme views on certain food groups without justification (i.e., in the absence of allergies or intolerances) may contribute to disordered eating attitudes and behaviors, especially in this period of psychological distress [27]. As diet and mood can mutually influence each other through the gut–brain axis [45], this appears to be even more urgent to address with correct information. The “clean eating” trend, in fact, may contribute to misinformation amongst the general public. Given the far-fetched health benefits claimed by its proponents, it could contribute to further confusion about nutrition and, thus, impact population health. On the other side, these results represent an opportunity to value the role of consumer food psychology in understanding and providing guidance for the citizens. It is clear that people want to gain back their decisional and psychological power over the food choices; the COVID-19 emergency could be the perfect occasion to restore a dialogue between the citizens, institutions, and food companies, which can address their need for reassurance and education.

## Figures and Tables

**Table 1:** Demographic profiles of the sample (*n* = 963).

	*n*	%	% Population
1. Gender			
Male	478	49.6	49.3
Female	485	50.4	50.7
2. Age			
18–24	95	9.8	10.0
25–34	155	16.1	16.3
35–44	209	21.7	21.5
45–54	218	22.6	22.7
55–59	106	11.0	10.8
60–70	95	9.8	18.8
3. Education			
Elementary	4	0.4	-
Junior high	129	13.4	-
Senior high	562	58.3	-
College or university	269	27.9	-
4. Geographic area			
North-West	253	26.3	26.3
North-East	180	18.7	18.6
Centre	188	19.5	19.7
South and Islands	342	35.5	35.5
5. Inhabited centre size			
Until 10,000 inhabitants	310	32.2	32.1
10/100,000 inhabitants	424	44.0	44.0
100/500,000 inhabitants	105	10.9	10.9
More than 500,000	124	12.9	12.9
6. Profession			
Entrepreneur/freelancer	121	12.5	12.4
Manager/middle manager	36	3.7	3.8
Employee/teacher/military	181	18.8	19.2
Worker/shop assistant/apprentice	206	21.4	21.0
Housewife	144	14.9	15.0
Student	48	5.0	5.3
Retired	77	7.9	7.9
Unoccupied	151	15.6	15.4
7. Household income level			
Until 1200 €	214	22.2	-
1201–1800 €	215	22.3	-
1801–3500 €	293	30.3	-
More than 3501 €	90	9.4	-
Missing	152	15.8	-

**Table 2:** The frequency of purchase of lactose-free and gluten-free food products (*n* = 963).

	*n*	%	Mean (SD)
In the last month, how many times did you purchase gluten-free food products?			2.18 (±1.29)
Never purchased (1)	419	43.5	
(2)	154	16.0	
(3)	177	18.4	
(4)	121	12.5	
Always purchased (5)	55	5.7	
I do not know	37	3.9	
In the last month, how many times did you purchase lactose-free food products?			2.29 (±1.33)
Never purchased (1)	381	39.5	
(2)	182	18.9	
(3)	150	15.5	
(4)	152	15.8	
Always purchased (5)	64	6.7	
I do not know	34	3.6	

**Table 3:** Frequency distribution of items.

	*n*	%	Mean (SD)	K	A
**Risk susceptibility** (N = 963)			3.32 (0.94)	−0.01	−0.36
Low (1–2)	157	16.2			
Medium (3)	382	39.6			
High (4–5)	425	44.1			
**Level of anxiety** (N = 963)			38.43(10.44)	−0.52	0.55
Normal	455	47.2			
Mild	292	30.3			
Moderate	186	19.3			
Severe	31	3.2			
**Level of depression** (N = 963)			41.07 (10.41)	−0.59	0.12
Normal	433	45.0			
Mild	212	22.0			
Moderate	258	26.8			
Severe	60	6.3			

Note: SD = standard deviation; K = Kurtosis; A = Asymmetry.

**Table 4:** Groups’ comparison on socio-demographic ad psychological variables.

	Consumers of Lactose-Free and Gluten-Free Products (*n* = 277)	Consumers of Lactose-Free Products (*n* = 179)	Consumers of Gluten-Free Products (*n* = 139)	Non Consumers (*n* = 326)	Total Sample (*n* = 921)	Pearson *X2*/F-Value	Effect Size η^2^	*p*-Value
Psychological variables (means)								
Anxiety	42.31 ^a^	38.93 ^b^	39.78 ^a,b^	34.28 ^c^	38.43	34.55	0.10	<0.001
Depression	43.65 ^a^	42.30 ^a^	42.92 ^a^	37.29 ^b^	41.02	23.83	0.07	<0.001
Risk-susceptibility	3.50 ^a^	3.27 ^a,b^	3.23 ^b^	3.20 ^b^	3.31	5.90	0.02	<0.01
Sociodemographic variables (%)								
Mean age (years)	45.20 ^a^	43.18 ^a^	41.96 ^a^	48.76 ^b^	45.58	10.70	0.03	<0.001
Gender						1.83	-	0.61
Male	48.0	49.2	46.0	52.1	49.4			
Female	52.0	50.8	54.0	47.9	50.6			
Level of education						0.94	-	0.82
Non-graduated	72.6	74.9	72.7	70.9	72.4			
Graduated	27.4	25.1	27.3	29.1	27.6			
Income level						6.44	-	0.09
Low (<1800€)	47.0	55.8	59.8	54.1	53.0			
High (>1800€)	53.0	44.2	40.2	45.9	47.0			

Note: η^2^ = eta squared; Different superscripts indicate significantly different means following ANOVA post hoc Bonferroni test.

## Data Availability

Data are fully available upon request to the corresponding author Greta Castellini, greta.castellini@unicatt.it.
